# Women’s attitudes, experiences and compliance concerning the use of Mindfetalness- a method for systematic observation of fetal movements in late pregnancy

**DOI:** 10.1186/s12884-017-1548-5

**Published:** 2017-10-16

**Authors:** Anna Akselsson, Susanne Georgsson, Helena Lindgren, Karin Pettersson, Ingela Rådestad

**Affiliations:** 1 Sophiahemmet University and Department of women and Child’s Health, Karolinska Institutet, PB 5605, S-114 86 Stockholm, Sweden; 2 Sophiahemmet University and Department of Clinical Science, Intervention and Technology, Karolinska Institutet, Stockholm, Sweden; 3 Department of Women and Child’s Health, Karolinska Institutet, Stockholm, Sweden; 4 Department of Clinical Science, Intervention and Technology, Karolinska Institutet, Stockholm, Sweden; 5 Sophiahemmet University, Stockholm, Sweden

**Keywords:** Fetal movements, Mindfetalness, Attitude, Compliance, Relationship

## Abstract

**Background:**

Maternal perception of decreased fetal movements and low awareness of fetal movements are associated with a negative birth outcome. Mindfetalness is a method developed for women to facilitate systematic observations of the intensity, character and frequency of fetal movements in late pregnancy. We sought to explore women’s attitudes, experiences and compliance in using Mindfetalness.

**Methods:**

We enrolled 104 pregnant women treated at three maternity clinics in Stockholm, Sweden, from February to July of 2016. We educated 104 women in gestational week 28–32 by providing information about fetal movements and how to practice Mindfetalness. Each was instructed to perform the assessment daily for 15 min. At each subsequent follow-up, the midwife collected information regarding their perceptions of Mindfetalness, and their compliance. Content analyses, descriptive and analytic statistics were used in the analysis of data.

**Results:**

Of the women, 93 (89%) were positive towards Mindfetalness and compliance was high 78 (75%). Subjective responses could be binned into one of five categories: Decreased worry, relaxing, creating a relationship, more knowledge about the unborn baby and awareness of the unborn baby. Eleven (11%) women had negative perceptions of Mindfetalness, citing time, and the lack of need for a method to observe fetal movements as the most common reasons.

**Conclusion:**

Women in late pregnancy are generally positive about Mindfetalness and their compliance with daily use is high. The technique helped them to be more aware of, and create a relationship with, their unborn baby. Mindfetalness can be a useful tool in antenatal care. However, further study is necessary in order to determine whether the technique is able to reduce the incidence of negative birth outcome.

## Background

The movements of the fetus as well as the mother’s experiences of the movements are unique to every fetus and pregnancy. The mother’s ability to perceive fetal movements is affected by factors such as gestational age, parity, obesity and the localization of the placenta [1, 2]. The greatest frequency of fetal movements is experienced when the women are lying down and a majority of women perceive most movements in late evening [3, 4].

Maternal concerns about fetal movements are the most frequent reason for unscheduled antenatal visits. In various populations between four and 15% of pregnant women will contact health care providers with concerns about fetal activity [5]. Decreased fetal movement can be a symptom of reduced oxygen and nutrient supply to the fetus via the placenta and the expected baby is at risk of being born prematurely, being born too small for gestational age, developing complications due to the lack of oxygen and even death [6].

The number of stillborn babies has remained at almost the same level in Sweden over the past 30 years. Four of 1000 children were born dead in 2014. The incidence has shown no tendency to decrease. In Sweden 2014, 463 babies were stillborn after gestational week 22, and another 175 babies died within 28 days after birth [7]. Maternal perception of decreased fetal movements is subjective and commonly used to assess fetal well-being [8]. Studies indicate an association between a low awareness of the fetal movements and negative pregnancy outcomes [9].

In a study by McArdle et al. [10] it was found that pregnant women prefer to receive as much information as possible about fetal movements. However, only 67% stated that they had received such information. The majority requested further information from their midwife or health care provider and additional written material to refer to at any time. In antenatal care in Sweden, until recently there has been a lack of national guidelines regarding how and when the midwife should inform the pregnant women about fetal movements; local guidelines have also varied. In October 2016, The National Board of Health and Welfare proposed national recommendations suggesting that all pregnant women should be informed about fetal movements on a routine visit to their midwife in gestational week 24. This information should include recommendations about when to seek health care if the woman has concerns due to decreased or weaker fetal movements [11]. However, there is no recommendation concerning the use of observational methods to increase the woman’s awareness of the movements. A previous study [12] indicates that women in Sweden are positive about observing fetal movements systematically. More knowledge about women’s experiences of using methods for observing fetal movements and how well they comply with the methods is needed in order to provide general recommendations to pregnant women.

Mindfetalness is a self-assessment method that the pregnant woman may use daily for getting to know the fetal-movement pattern. Mindfetalness can strengthen the woman’s awareness of the unborn baby, increasing the possibility for a healthy baby being born [13].

The aim of this study was to explore women’s attitudes, experiences and compliance concerning the practice of Mindfetalness in late pregnancy.

## Method

### Study design

This study was carried out at three maternity clinics in Stockholm, Sweden, between 15th of February and 7th of July, 2016. The clinics provide care to about 670 pregnant women per year. Initially the seven midwives working at the clinics were informed about the study and received a lecture about the observation method Mindfetalness [13].

### The intervention

Mindfetalness should be practiced for 15 min daily when the fetus is awake and, if possible, the woman should lie on her left side when she focuses upon her unborn baby’s movements. By lying on her left side the flow of blood in the placenta in order to avoid Vena cava syndrome is facilitated [14]. When practicing Mindfetalness, the woman focuses upon the intensity and character of the movements, as well as the frequency, without counting them. The recommended starting point is gestational week 28 [13].

### Participants

Inclusion criteria for participating in the study were the ability to understand Swedish and to be following the standard antenatal care program. The midwives consecutively distributed written information about Mindfetalness to women, who visited the clinic during pregnancy week 28–32. During the study period 104 women received information. The information included general information about fetal movements and how to practice Mindfetalness. When the pregnant woman received the written information about the method the midwife also verbally gave instructions about how to perform Mindfetalness. Participation was voluntary. On subsequent visits to the midwife the women were asked to respond verbally to the following questions (both closed and open-ended): “What did you think about the method Mindfetalness?”, “Are you positive or negative to the method?”, “Have you used the method?” and “If so, how often and for how long?”. The midwife noted the women’s answers. Data regarding age, nationality, parity and education level were collected from the medical records and the Swedish Pregnancy Register.

### Analysis

Age, nationality, parity and education level were analysed in order to investigate differences in attitudes to the method. Analysing compliance (using the method daily) started after the first follow-up and continued until the woman gave birth. Compliance with the method was compared with the group’s parity. Fisher’s exact test was used to compare groups and a *p*-value of < 0.05 was considered statistically significant. The women’s answers to the question, “What did you think about the method Mindfetalness?” were analysed using a qualitative manifest content analysis [15, 16]. To obtain an overall picture, the comments were read repeatedly. Since the material consisted of short sentences documented by the midwife, verbatim quotations were used in the analysis. Preliminary themes were identified and the material was organized. Each quotation was placed in preliminary categories. In the third phase, one code at a time was surveyed and all the quotations in the code analysed and placed in categories. Four members of the research team then read the quotations and the organized material. This process led to the final categories [15, 16]. The study was approved by The Regional Ethic committee in Stockholm, Sweden, Dnr 2015/2105–31/1.

## Results

A total of 104 pregnant women participated in the study. The women were 17–42 years old and about the same number of nulliparous as multiparous women. Further, most of the women had higher education and the majority were born in Sweden. There was no statistical significant difference in women’s attitudes towards Mindfetalness with regard to age, nationality, parity and education level (Table 1).

**Table 1:** Attitudes to the method of Mindfetalness related to women’s age, parity, educational level and country of birth

	Positive attitude to Mindfetalness n (%)	RR	CI	*P*-value
Age				
17–24	3/3 (100)	1.2	Not done^b^	1.0
25–34	61/72 (85)	Reference	Reference	Reference
35–42	28/29 (97)	1.1	1.0–1.3	0.2
Parity				
Primipara	43/48 (90)	Reference	Reference	Reference
Multipara	49/56 (88)	1.0	0.9–1.1	1.0
Education^a^				
≤ Elementary school	10/12 (83)	0.9	0.7–1.1	0.2
High school	27/28 (96)	Reference	Reference	Reference
College/university	53/60 (88)	0.9	0.8–1.0	0.4
Country of birth				
Sweden	67/78 (86)	Reference	Reference	Reference
Not born in Sweden	25/26 (96)	1.1	1.0–1.3	0.4

^a^missing *n* = 4 (3 Education unknown whereof 3 positive/1 negative)

^b^Not done due to small numbers

### Attitude

Almost all, 93 (89%) of the women had a positive attitude to the method Mindfetalness. Eleven (11%) were negative but one woman changed her attitude from negative to positive during her pregnancy. Lack of time (*n* = 6), not being in need of a method to observe fetal movements because the baby was so active (*n* = 3) were reasons for being negative. One woman did not like the structured way of focusing upon movements and one said that she thought that the method would cause more worry.

The women who were initially positive towards Mindfetalness were also positive about the method throughout the pregnancy.

### Experiences

Five categories were identified in the analyses of the women’s experiences of practicing Mindfetalness: Knowledge about the unborn baby, Awareness of the unborn baby, Creating a relationship with the baby, Decreased worry and Relaxing.

#### Knowledge about the unborn baby

In this category the women described feelings of increased knowledge about the movement pattern. Also, they stated that they became more aware about their unborn baby in general.

*“I get to know the baby’s pattern”.*

*“I lie on my back instead of on the side, otherwise the baby protests because she/he doesn’t like the side”.*

#### Awareness of the unborn baby

In the category, *Awareness of the unborn baby,* the women reported that Mindfetalness was a good method for having control over their baby and also for giving time to the unborn baby. Some women said that they discovered new things about their baby and that they became more aware of the baby as an individual.

*“This made me more aware of the baby and I started to think about small details in a positive way”.*

*“I was surprised when I did this, that the baby is an individual. Before, it felt more fuzzy”.*

#### Creating a relationship with the baby

In this category the women described that they had become more acquainted with their unborn baby. Practicing Mindfetalness had increased their awareness of the baby’s individuality and helped them build a relationship. Further, some of the women reported that family members joined them when they practiced Mindfetalness and that they also “listened” to the unborn baby.

*“I′m building a relationship”..*

*“My husband is also with me and listens, he has his hands on my tummy during this time”.*

#### Decreased worry

In the category *Decreased worry* some women stated that they felt more confident and not so worried about fetal movements. Further, they described using Mindfetalness as a tool if they felt worried.

*“I was worried about fetal movements before but I′m not anymore because of the information that I received and by practicing the method Mindfetalness”.*

*“I practice the method more when I get worried about fetal movements. Now, I’m not as worried as before”.*

#### Relaxing

Some women thought that Mindfetalness led to a ‘cosy moment’ and helped them to relax.

*“Mindfetalness is a cosy thing, it helps me to relax”.*

*“It’s a good way to wind-down, relaxing”.*

### Compliance

Eighty-seven (84%) of all women in this study practiced Mindfetalness (90% of the women who were positive to the method) and most practiced it daily for the suggested duration i.e. 15 min. Seventy-eight (75%) women used the method daily after the first follow-up (64% of the multiparous and 88% of nulliparous women). Among the women that stated they were positive towards the method compliance was high (98% of the nulliparous women and 72% of the multiparous women). As presented in Table 2, nulliparous women used the method to a greater extent than multiparous (*p* = 0.007). Five women stated that they stopped using the method in the prescribed way (laying down on the left side for approximately 15 min). They reported that the method had become a routine and that they practiced their own form of Mindfetalness automatically every day and sometimes several times a day.

**Table 2:** Compliance: nulliparous compared with multiparous women

Parity	Practice Mindfetalness daily n (%)	RR	CI	*p*-value
Primipara	42/48 (88%)	1.4	1.1–1.7	0.007^a^
Multipara	36/56 (64%)	Reference	Reference	Reference

^a^Statistic significant

## Discussion

Most women had a positive attitude towards Mindfetalness. When practicing Mindfetalness they felt they gained more knowledge about, increased their awareness of, and created a relationship with their unborn baby. Further, they felt less worried and more relaxed. The women’s compliance with the method was high and most of them practiced Mindfetalness daily until birth.

Our finding, that the women had a positive attitude to Mindfetalness, is in accordance with a crossover trial by Malm et al. [12] where 40 pregnant women tested both Mindfetalness and the count-to-ten method, i.e. they measured the time it took to feel ten movements. Most women preferred Mindfetalness to the count-to-ten method when they were asked to choose between the two.

In other studies [17, 18] comparing different methods of counting fetal movements, the “count-to-ten” method was found to be the one most favoured by pregnant women. In a study by Saastad et al. [19], 1013 pregnant women were randomly assigned to perform daily fetal movement counting or standard care (not counting the movements). Seventy-nine per cent responded favourably to the use of counting charts. Using a method for systematic observation of fetal movements can be a useful tool in helping women focus upon the expected baby. Some women may prefer counting the movements and some might prefer to focus upon the quality of the movements.

Our data indicate a tendency for the oldest women to have a more positive attitude to Mindfetalness than the younger. Women aged 35–42 may be more positive towards the method due to their knowledge that the risks during pregnancy increase with age. High levels of anxiety are common in the advanced age group as well as a higher perception of pregnancy risks [20, 21].

A minority of the women in this intervention study stated that they were negative towards Mindfetalness and the main reasons were that they did not have time. More often multiparous women stated that they had lack of time. In Sweden the female employment rate is high (78%) and women are working to the same extent as men [22]. A pregnant woman who has got children to pick up at preschool after a day’s work might have less time to follow the method than nulliparous. Further almost all the women who were positive about the method but did not practice Mindfetalness were multiparous. It might be that they feel more comfortable and know what to expect regarding fetal movements. Some multiparous women said that they believed the method to be favourable for nulliparous women. Studies have shown that nulliparous women tend to seek healthcare due to concerns about decreased fetal movements to a greater extent than multiparous women [6].

Mindfetalness were generally a positive experience for the women in our study. The method led to more knowledge and awareness of the unborn baby and it also helped the women to create a relationship with the baby. Further, the women felt calm and less worried when they practiced the method. These findings are in line with Malm et al. [12]. The women in their study felt calm, relaxed, focused and mentally present when they practiced the self-assessment methods. A study by Raynes-Greenow et al. [23] examined maternal perception of fetal movements using a qualitative framework. They suggest that women’s perception of fetal wellbeing based on their own assessment of fetal movement is a high priority in clinical research. As far as we know, apart from Malm et al. [12], no other studies have evaluated a method that does not focus upon kick counting. In a study by Draper et al. [24], 23% of women completing a fetal movement chart became more worried and RCOG guidelines from 2011 [1] suggest that pregnant women can become more worried by receiving information and being instructed to count movements. This is opposite to the findings in a study by Saastad et al. [19] showing that those women who counted the movements felt less worried, got to know their child and felt it reassuring to use the method. Further, in another study [25] Saastad el al. showed that women who performed fetal movement counting from gestation weeks 28 – 37 reported less anxiety than those in the control group. However, the discrepancy in data concerning worry can be interpreted as for some women the counting-method creating anxiety because of the counting itself which does not seem to be the case with Mindfetalness. Counting focuses upon numerical results whereas Mindfetalness focuses upon what the woman feels.

Our findings indicate that Mindfetalness can help the woman and other family members to strengthen their relationship with the unborn baby. The women in our study stated that they were creating a relationship with their unborn baby when practicing Mindfetalness. In one study [26], where women were asked if they felt daily periods of much fetal movement it emerged that women who experienced several occasions with a lot of movement rated higher on a scale measuring prenatal bonding compared to women with few such occasions. Our findings can also be confirmed by the study of Mikhail M. S. et al. [27] showing that a woman’s awareness of fetal movements may enhance the maternal-fetal attachment. A later study by Saastad, et al. [28] did not find this connection. However, these two studies evaluated fetal movement counting which was not the case in the present study.

The high compliance with systematic self-assessment of fetal movements are confirmed by other studies [18, 28, 29]. The count-to-ten method seems to be the most preferred self-assessment method that has been tested [17, 18]. In a study by Georgsson et al. [30] 1000 women who consulted healthcare due to decreased fetal movements completed a questionnaire regarding what they wanted to communicate to other pregnant women and to health care professionals who take care of women with decreased fetal movements. The recommendation was that other pregnant women should pay attention to fetal movements. The high compliance with monitoring fetal movements is probably due to pregnant women having a generally high awareness of the importance of fetal activity, which to some extent is affected by the important information from the midwife in Swedish antenatal care.

The concept of Mindfetalness was in this study always presented together with basic information about fetal movements. The women described the method as a useful tool and said that they read the written information they received several times, which they found supportive. Some women reported using Mindfetalness as a tool when they became worried about fetal movements. Further, some women recorded their thoughts in a dairy, to which the written information was attached, whereas some women made notes on their mobile telephone. Other studies confirm that pregnant women want information about fetal movements. The majority of the women in a study by McArdle et al. [10] requested information about fetal movements from their midwife and also asked for printed material to refer to at any time. Further, in the study by Georgsson et al. [30] women asked for improved and uniform information about fetal movements. The women wanted to receive this information from their midwife, which is in line with other studies [10, 31, 32]. In these studies women completed questionnaires about the most important source of information during pregnancy and the midwife was chosen as the most important in all three studies.

### Methodological considerations

The sample in the study is small. Further, all women were Swedish speaking and the study was conducted in one area of Stockholm, the capital of Sweden. Despite this, and with no ambition to generalize to a non- Swedish speaking population, we do think this study gives us new knowledge about women’s attitudes to, and compliance with, Mindfetalness.

The pregnant woman builds a relationship with her midwife during pregnancy, she listens to, follows advices and often relies upon the midwife. Thus, the midwife plays an important role and this can explain the positive attitudes towards, and high compliance with the method in this study. Almost all women in Sweden visit a midwife during their pregnancy and antenatal care is free of charge. The midwife is the sole care provider if the pregnancy proceeds normally. [33].

Luyben and Fleming [34] summarizes after an interview study among pregnant women that “establishing a sharing trust relationship” is important for antenatal care. This can also to some extent be one of the study’s limitations, i.e. that the woman does not always choose to give an accurate report to the midwife regarding compliance with the method.

A limitation in the qualitative data collection is that the midwife only wrote down the main points of what the women said and then verbally reported this to the researcher. This therefore means that the midwife’s perception of the woman’s thoughts is included in the data.

## Conclusion

Women in late pregnancy were in general positive to Mindfetalness. The women experienced increased knowledge and more awareness of the unborn baby. Further, the method helped them to create a relationship with the unborn baby and they also felt less worried and more relaxed. Compliance with making daily observations of the fetal movements using Mindfetalness was high.

### Clinical implications

Mindfetalness can be a useful tool in antenatal care for making systematic observations of fetal movements. Further research is needed to examine the effects of Mindfetalness on fetal wellbeing.
